# Systematic review on the effectiveness of mobile health applications on mental health of breast cancer survivors

**DOI:** 10.1007/s11764-023-01470-6

**Published:** 2023-10-31

**Authors:** Anna Schäfer, Steffi Jírů-Hillmann, Jonas Widmann, Felipe A. Montellano, Jessica Salmen, Rüdiger Pryss, Achim Wöckel, Peter U. Heuschmann

**Affiliations:** 1 Institute of Clinical Epidemiology and Biometry, Julius-Maximilians-Universität Würzburg, Würzburg, Germany; 2 Department of Neurology, University Hospital Würzburg, Würzburg, Germany; 3 Department of Gynecology and Obstetrics, University Hospital Würzburg, Würzburg, Germany; 4 Institute for Medical Data Science, University Hospital Würzburg, Würzburg, Germany; 5 Clinical Trial Centre Würzburg, University Hospital Würzburg, Würzburg, Germany

**Keywords:** Breast cancer, Follow-up care, Mental health, mHealth, Systematic review

## Abstract

**Purpose:**

Breast cancer survivors are more likely to report psychological distress and unmet need for support compared to healthy controls. Psychological mobile health interventions might be used in follow-up care of breast cancer patients to improve their mental health.

**Methods:**

We searched MEDLINE, PsychINFO, Cochrane and PROSPERO for articles on controlled trials examining the effectiveness of psychological mobile health interventions compared to routine care regarding mental health outcomes of adult breast cancer survivors. This review followed the PRISMA statement and was registered on PROSPERO (CRD42022312972). Two researchers independently reviewed publications, extracted data and assessed risk of bias.

**Results:**

After screening 204 abstracts published from 2005 to February 2023, eleven randomised trials involving 2249 patients with a mean age between 43.9 and 56.2 years met the inclusion criteria. All interventions used components of cognitive behavioural therapy. Most studies applied self-guided interventions. Five studies reported percentages of patients never started (range = 3–15%) or discontinued the intervention earlier (range = 3–36%). No long-term effect > 3 months post intervention was reported. Three of seven studies reported a significant short-term intervention effect for distress. Only one study each showed an effect for depression (1/5), anxiety (1/5), fear of recurrence (1/4) and self-efficacy (1/3) compared to a control group.

**Conclusions:**

A wide variance of interventions was used. Future studies should follow guidelines in developing and reporting their mobile interventions and conduct long-term follow-up to achieve reliable and comparable results.

**Implications for cancer survivors:**

No clear effect of psychological mobile health interventions on patients’ mental health could be shown.

**Registration:**

PROSPERO ID 312972.

**Supplementary Information:**

The online version contains supplementary material available at https://doi.org/10.1007/s11764-023-01470-6.

## Introduction

Breast cancer is the most frequent cancer in women worldwide, but due to advances in treatment options, the mortality rates for breast cancer are decreasing, especially in developed countries, thus increasing the number of survivors [1, 2]. Breast cancer survivors are at higher risk for mental health impairments such as stress, anxiety or depression compared to healthy controls [3]. However, several studies report unmet psychological needs among breast cancer patients, and waiting times for psychotherapy can be very long [4–6]. While screening for psycho-oncological needs during the acute diagnosis and treatment phase is common, few studies focus on survivorship [4, 7–9]. Thus, breast cancer survivors, especially after primary treatment, might not receive adequate psychological follow-up care.

Mental health support can potentially be expanded through electronic or mobile health applications (eHealth or mHealth). In terms of eHealth, information processing and distribution happens electronically to promote and support patient treatment, which, thus, also includes internet-based interventions [10, 11]. mHealth can be subsumed under eHealth and refers to services that run via mobile devices. Psychological eHealth interventions are not only effective for treating mental disorders, but also in its prevention or reduction of symptom scores [10, 12]. They can be distinguished between self-help offerings, where the patient receives the digital intervention without being in contact with the health care provider while using the app, and guided interventions, where there is also the possibility to contact and receive feedback from the health care provider via the application [10]. Interventions are mostly based on a theoretical foundation (e.g., cognitive behavioural therapy (CBT), health behaviour or psychodynamic models) and can be used in prevention, treatment and follow-up care. Additionally, it is recommended that the health literacy and needs of patients are considered in the development of interventions [13].

Research on psycho-oncological care approaches identified some studies with effects of psychological eHealth interventions for cancer patients in general, recommending their use to reduce psychological distress, depression, anxiety and fatigue regardless of the level of distress, but the results are not as clear as those of psycho-oncological eHealth interventions on quality of life [14]. Previous systematic reviews on eHealth and mHealth report heterogeneity across studies in terms of applications, patient groups and psychometric assessment of mental health [15–17]. In a systematic review of mobile apps for breast cancer patients that was published 6 years ago, no app with psychological intervention was found [18].

This systematic review therefore aims to identify and examine previously published controlled trials to examine the effectiveness of psychological mHealth interventions compared to routine care in improving mental health outcomes such as distress, depression, anxiety and self-efficacy of breast cancer survivors after primary treatment.

## Methods

### Characteristics of the systematic review

We followed the Cochrane suggestions for systematic reviews and the PRISMA Statement for reporting its methods and results [19, 20]. Our study protocol was registered on PROSPERO in February 2022 (ID 312972).

### Search strategy

The aim of this systematic review was to assess the effectiveness of mobile or web-based applications for the psycho-oncological follow-up care of breast cancer patients considering mental health in the short term (≤ 3 months post intervention) and long term (> 3 months post intervention). We defined our research question and conducted our search based on a priori defined PICOS criteria (population, intervention, comparison, outcome, study design). We included studies with a population of adult breast cancer survivors (after primary treatment: surgery, chemotherapy, radiation), in curative treatment. Studies that examined a broader population of cancer patients were asked if they could provide data for the breast cancer survivor subgroup and were otherwise excluded. Regarding the intervention, we focused on mobile applications, also including web-based psychological interventions that can be accessed with a mobile device (“psychological mHealth interventions”). Psychological interventions were defined as non-pharmacological, psychological-oriented therapeutic approach with proposed mechanisms on mental health, either consisting of an evidence-based psychotherapy or minimally psychotherapeutic components like psychoeducation, relaxation, motivation analysis or coping skills training [12, 16]. Interventions only consisting of support groups or focussing on somatic symptoms or lifestyle factors such as nutrition, weight or physical exercises were excluded. Trials with more than two treatment-arms were included and the treatment arm with psychological mHealth intervention only as well as the arm with routine care as control arm were included. Trials with usual care, waitlist or an active control group were eligible. The following outcomes had been defined as relevant for the scope of our review [3, 12, 15, 18, 21, 22]: psychological distress (e.g. measured via distress thermometer [23], PSS-10 [24] or BSI [25]), anxiety (e.g. via HADS [26, 27] or GAD-7 [28]), depression (e.g. via HADS or PHQ-9 [29]) and self-efficacy (e.g. via CSE [30] or CBI [31]). Cancer-related fear as an important outcome related to breast cancer patients’ mental health (measured via fear of recurrence/progression scales) was also a possible outcome. Finally, we included the following study-designs: (randomised) controlled or clinical as well as pilot trials.

Searches of the electronic databases MEDLINE (via pubMED), PsychINFO (via ProQuest), Cochrane Database of Systematic Reviews, Cochrane Central Register of Controlled Trials (CENTRAL, via Cochrane Library) and PROSPERO were carried out on 13 March 2022 and updated on 21 February 2023. No publication time or language restrictions were applied during the search. The search strategy, including a combination of medical subject headings and free text terms, is listed in detail in the supporting information (Table S1). In addition, a manual search was conducted based on references within publications identified.

### Study selection

**Fig. 1:**
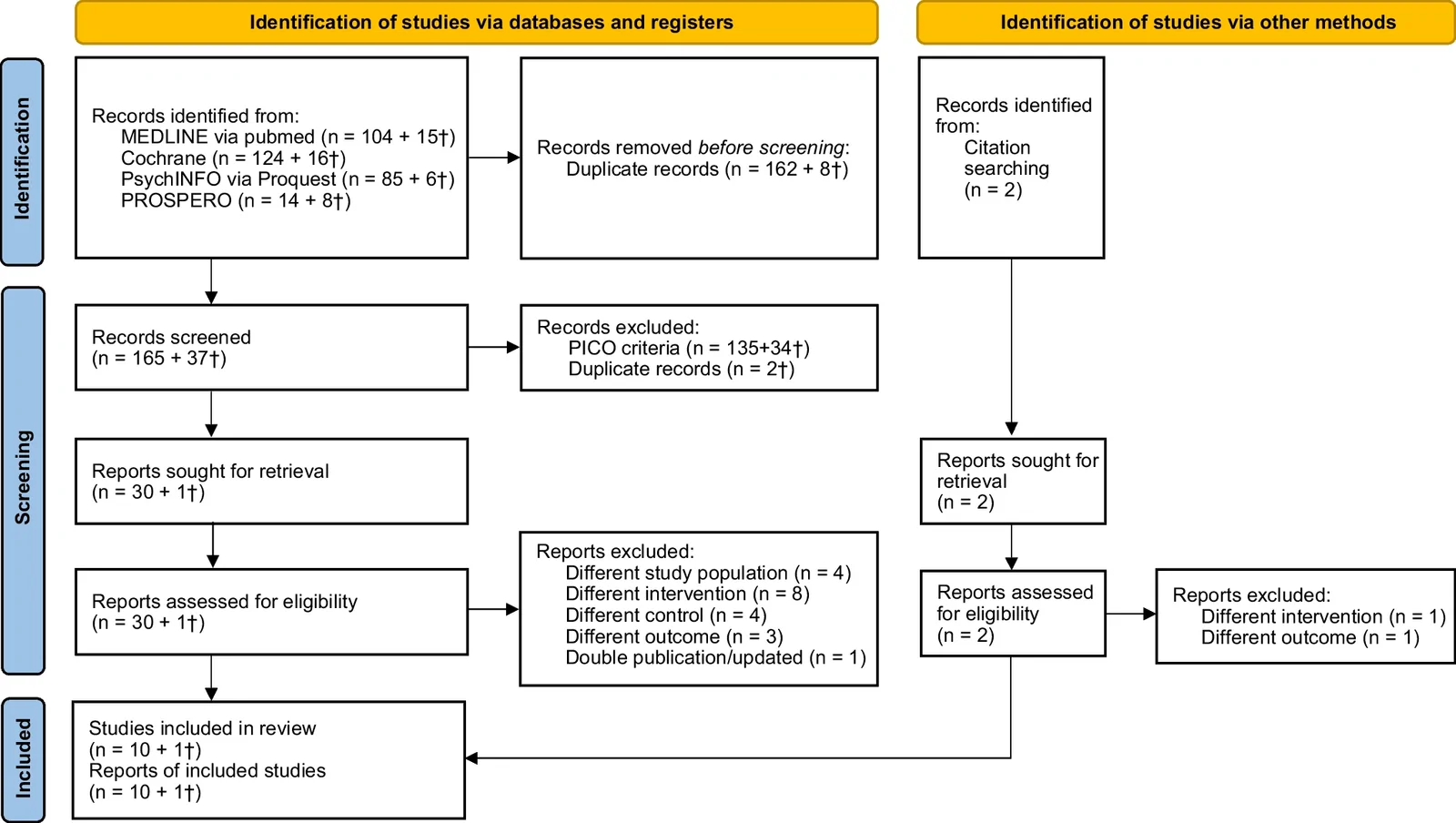
PRISMA 2020 flow diagram [66]. ^†^Updated search

We exported the results of the systematic search into the Endnote Reference Manager and removed duplicates both automatically and manually. Two out of a team of four reviewers (AS, SJH, JW and FAM) independently assessed the abstracts and titles of each publication for relevance according to the pre-defined PICOS criteria. Subsequently, two reviewers independently screened the full-text articles for eligibility. For each publication, the two reviewers resolved any disagreement through discussion or through decision by a third reviewer. The exclusion of full-text articles based on defined criteria was documented (Fig. 1).

### Quality assessments of the studies

Two reviewers independently reviewed the remaining studies using the checklist of the National Institute of Health Risk Assessment Work Group [32] and an additional question regarding external validity from the Critical Appraisal Skills Programme [33]. The publications were evaluated according to their risk of bias, the attribution of study results to the intervention, statistical analysis and the applicability of results. Any disagreement was resolved by argumentative exchange or through decision by a third reviewer.

### Data extraction and analysis

Two researchers extracted the relevant information and results for every publication. We extracted results from both short-term and long-term follow-up assessments (≤ / > 3 months post intervention). The results were analysed qualitatively. For a possible meta-analysis, we checked whether there were enough homogeneous studies defined by meeting the following four criteria: at least three trials with the same endpoint; with similar timing of the outcome assessment; similar intervention and control group; and a methodological quality level that was rated of at least fair quality.

## Results

### General characteristics of selected studies

We retrieved 327 records from the initial search carried out in March 2022. After deduplication, we screened the titles and abstracts of the remaining 162 publications. Subsequently, we obtained 30 full texts and assessed the studies according to the inclusion and exclusion criteria. Three potential matching studies looked at a broader population of cancer patients and were asked if they could provide data for the breast cancer survivor subgroup. We did not hear back from the authors of two studies, and we heard back from the authors of one study, but it was not possible to provide the data. We included 10 trials in this systematic review. The updated search from March 2022 to February 2023 revealed 45 publications, of which eight duplicates were removed before screening. One trial was included in full-text screening and included in the review (Fig. 1).

The eleven included trials examined 2249 patients with mean age ranging from 43.9 to 56.2 years (Table 1). Nine studies had female gender as an inclusion criterion; the two remaining studies reported no sex distribution. The median duration of follow-up was 3 months (range = 1–10 months). Two trials used active control conditions, consisting of access to the app or web-platform with general health content, but no psychological content [34, 35]. One trial compared three different intervention components using a 2^3^ × 2 design and Multiphase Optimization Strategy with only a small routine care control group [36]. The other eight trials compared the intervention group to routine care with or without waiting list [37–44].

**Table 1:** Characteristics of included trials examining psychological mHealth interventions for breast cancer survivors

First author, year, country	Study type	Number of participants at baseline/post-intervention/follow-up		Mean age (SD)		Breast cancer stage	Months since diagnosis	Intervention group				Control group
		Intervention	Control	Intervention	Control			Type	Duration in months	Theory	Guidance	
Abrahams, 2017, The Netherlands	RCT (multicentre)	66/66	66/64	52.5 (8.2)	50.7 (7.6)	I (48%) II (38%) III (14%)	Intervention: 43.7 (31.0) Control: 39.0 (25.5)	Web	6	CBT	Therapist-guided	Routine care/waitlist
Admiraal, 2017, The Netherlands	RCT (multicentre, parallel-arm study)	69/63	69/64	53.1 (9.8)	53.2 (8.5)	I (46%) II (2.5%) III (52%)	Intervention: 8.7 (2.1) Control: 8.7 (1.9)	Web	3	Theory of problem solving, use of active approach-oriented coping	Self-guided (+ contact option)	Routine care/waitlist
Akechi, 2022, Japan	RCT (multicentre, two-arm)	223/220/213	224/224	43.9 (4.57)	44.0 (4.49)	0 (9.9%/6.3%) I (39.9%/37.5) II (39.9%/44.6%) III (6.7%/9.4%)	Years since surgery: 12–24 (43%) 24–48 (44%) > 48 (13%)	App	2	CBT, including problem-solving therapy (PST) and behavioural activation (BA)	Self-guided	Routine care/waitlist
Atema, 2018, The Netherlands	RCT (multicentre)	Guided intervention: 85/82/79 Self-managed: 85/80/77	84/80/80	Guided: 47.5 (5.1) Self-managed: 47.7 (5.7)	47.0 (5.5)	Not reported	37.2	Web	2	CBT	Self- and therapist-guided	Routine care/waitlist
Cinar, 2021, Turkey	RCT (single centre)	31/31	33/33	45.9 (8.3)	45.5 (9.8)	I-III	Disease duration: intervention: 30.5 (25.6) control: 29.7 (19.8)	App	3	Education and relaxation, symptom diary	Self-guided (+ contact option)	Routine care/waitlist
Holtdirk, 2021, Germany	RCT	181/141/126	182/165/137	50.1 (8.5)	49.8 (8.0)	Not reported	Not reported	Web	3 (access to Optimune for 1 year)	CBT (targeting depression, anxiety and fatigue as well as stress management and behaviour change)	Self-guided	Routine care/waitlist
Hummel, 2017, The Netherlands	RCT	84/69	85/82	51.6 (7.2)	50.5 (6.8)	Not reported	37.5 (16.3)	Web	6	CBT (mainly for sexual functioning and relationship)	Therapist-guided	Active control (waitlist with information booklet and phone calls)
Oswald, 2021, USA	Pilot RCT	40/37/37	40/38/36	53.5 (11.3)	51.6 (11.5)	0 (4%) I (36%) II (41%) III (14%)	Not reported	App	2	Stress and coping	Self-guided	Active control (My Health smartphone app)
van den Berg, 2015, The Netherlands	RCT (multicentre)	70/62/62/63	80/71/73/72	51.4 (8.3)	50.2 (9.15)	Not reported	Not reported	Web	4	CBT (four phases of adjustment)	Self-guided	Routine care/waitlist
van Helmondt, 2019, The Netherlands	RCT (multicentre)	128/109/91	128/120/107	55.3 (10.1)	56.2 (9.8)	Not reported	31.2	Web	3	CBT	Self-guided	Routine care/waitlist
Wagner, 2021, USA	2^3 × 2 (full factorial design using Multiphase Optimization Strategy (MOST))	172 ( at least one CBT component received)	24 (no CBT components received)	Relaxation 54.8 (10.2) Worry practice 54.4 (10.2) Cognitive restructuring 55.6 (9.5) Tele-coaching 55.3 (9.4)	Relaxation 54.8 (10.2) Worry practice 54.4 (10.2) Cognitive restructuring 55.6 (9.5) Telecoaching 55.3 (9.4)	0 (2.6%) I (44.4%) II (39.8%) III (13.3%)	2.4–229.2	Web	1	CBT (participants received 1–3 CBT components)	Self- and therapist-guided	Factorial design/mostly active control

*SD* standard deviation*, RCT* randomised controlled trial, *CBT* cognitive behavioural therapy

### Quality and risk of bias assessment

**Fig. 2:**
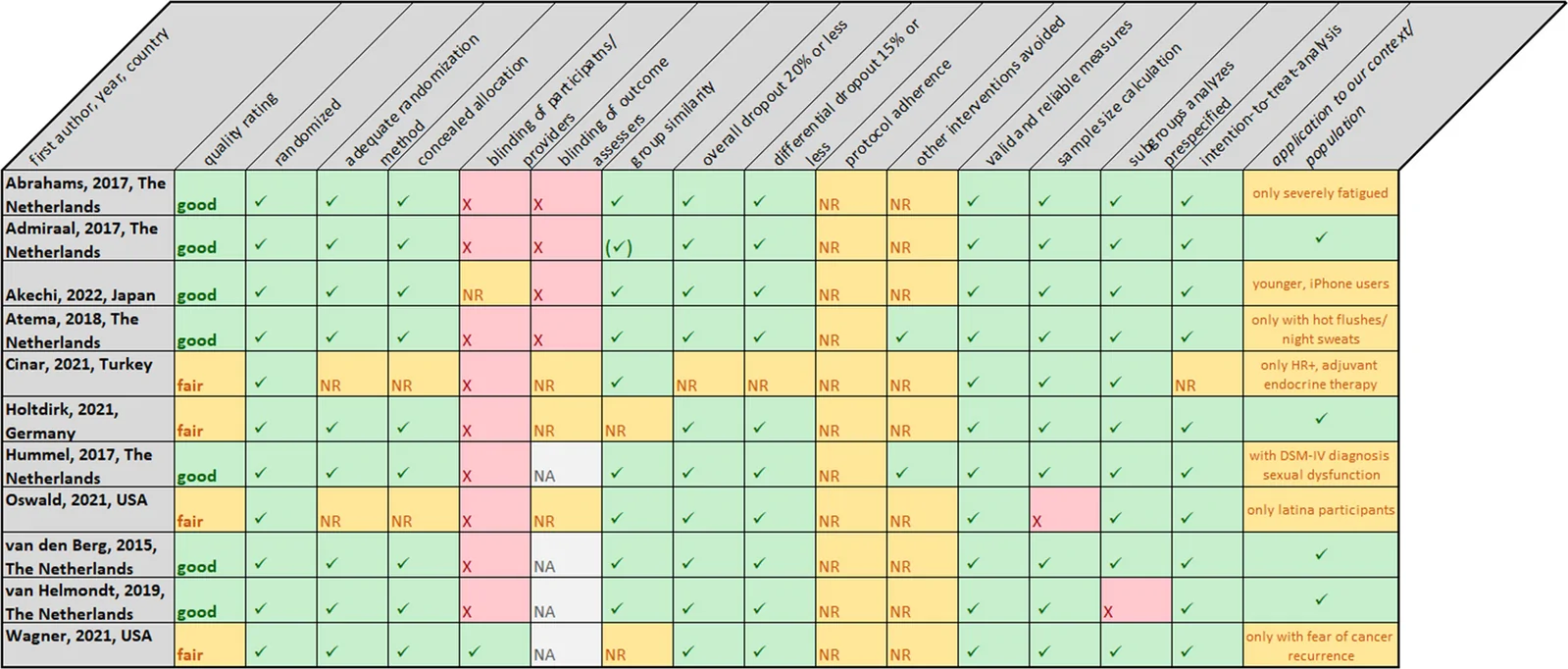
Quality rating according to the NIH Risk Assessment Work Group [32]. NR, not reported; NA, not applicable

Seven trials were classified as being of good quality, four were classified as being of fair quality (Fig. 2). It was noted that information was often missing on whether those assessing outcomes were blinded, whether the protocol was followed closely and whether other interventions were avoided.

### Meta-analysis

The requirements for meta-analysis were not met (enough homogeneous studies regarding outcome, intervention and control group with an adequate methodological quality). Therefore, results were analysed using narrative synthesis only.

### Intervention characteristics

All interventions were CBT-based. Eight interventions were generally based on CBT, the other three interventions combined two of the following components: psychoeducation, coping and stress management (Table 1). Seven studies provided information on the development of their mHealth intervention in the publication or in their study protocol. Patient involvement was mentioned four times, the implementation of a pilot phase three times and the consideration of health literature of the target group once. One app came from a commercial provider. Median duration of interventions was 3 months (range = 1–6 months). Of the eleven trials included in this review, eight were web-based and three used native apps. Five trials applied self-guided mHealth interventions, two used self-guided interventions with the option to contact a professional, two used therapist-guided mHealth interventions and another two studies compared self- and therapist-guided interventions. On the one hand, there was no difference in effectiveness between unguided and guided interventions in the included studies. On the other hand, two studies that directly compared guided and unguided interventions reported that usage rates were higher for the guided intervention compared to the unguided one [36, 40]. Five studies reported mean frequency or duration of intervention usage (Table 2).

**Table 2:** Summary of results of included trials examining psychological mHealth interventions for breast cancer survivors

First author, year, country	Follow-up after baseline in months	Primary outcomes	Secondary outcomes	Relevant outcome scale	Significant intervention effect		Intervention usage	Quality rating	Restrictions/applicability
					Short-term (≤ 3 months post intervention)	Long-term (> 3 months post intervention)			
Abrahams, 2017, The Netherlands	6.0	Fatigue severity	Functional impairment, psychological distress, quality of life	Brief Symptom Inventory-18	**Yes**: fatigue severity; functional impairment; psychological distress **No**: quality of life	No long-term follow-up	Not reported	Good	Only severely fatigued patients
Admiraal, 2017, The Netherlands	1.5 (mid-treatment) 3.0	Increased optimism and control over the future	distress, quality of life	Dutch distress thermometer, Increased optimism and control over the future subscale of constructs empowering outcomes questionnaire	**No**: increased optimism and control over the future; distress; quality of life	No long-term follow-up	Not reported	Good	
Akechi, 2022, Japan	2.0 6.0 (intervention group only)	Fear of cancer recurrence	Fear of cancer recurrence, anxiety and depression, care needs, post-traumatic growth, satisfaction with intervention	Fear of Cancer Recurrence Inventory-Short Form, Japanese Concerns About Recurrence Scale, Hospital Anxiety and Depression Scale	**Yes**: fear of cancer recurrence **No**: depression; anxiety *Greater improvement observed among those with high fear of cancer recurrence compared with those with low fear of cancer recurrence*	Only intervention group, changes from 2 to 6 months follow-up: **Yes**: depression **No**: other outcomes	App sessions completion: none to all available sessions; mean completion of 4.7 (SD = 1.9) for 6 available sessions and a mean completion of 6.7 (SD = 3.3) for 9 available sessions; estimated 10 minutes per session	Good	Only younger breast cancer survivors who were iPhone/iPad users
Atema, 2018, The Netherlands	2.5 6.0	Perceived impact of hot flushes/night sweat, overall levels of menopausal symptoms	Sleep quality, frequency of hot flushes/night sweat, sexual functioning, psychological distress, quality of life	Hospital Anxiety and Depression Scale total and subscales	**Yes**: perceived impact of hot flushes/night sweat; menopausal symptoms: (guided); sleep quality; night sweats frequency (guided) **No**: menopausal symptoms (unguided); hot flushes frequency; night sweats frequency (unguided); sexual functioning, psychological distress, quality of life	**Yes**: perceived impact of hot flushes/night sweat (guided); menopausal symptoms (unguided); sleep quality (guided); hot flushes frequency **No:** perceived impact of hot flushes/night sweat (unguided); menopausal symptoms (guided); sleep quality (unguided): night sweats frequency; sexual functioning, psychological distress, HRQoL	Not reported	Good	Only patients with treatment-induced problematic hot flushes/night sweats
Cinar, 2021, Turkey	3.0	Quality of life, distress (not specified if primary or secondary outcome)	Not reported	NCCN Distress Thermometer	**Yes**: quality of life; distress	No long-term follow-up	Not reported	Fair	Only patients with hormone receptor-positive cancer, treated with adjuvant endocrine hormone therapy
Holtdirk, 2021, Germany	3.0 6.0	Quality of life, physical exercise, dietary habits	Cancer-related fatigue, cancer-related emotional impact, depression, anxiety, fear of progression, insomnia symptoms, subjective usefulness of the programme	Generalized Anxiety Disorder Scale-7, Patient Health Questionnaire-9, Fear of Progression questionnaire 12 items	**Yes**: quality of life; dietary habits; insomnia; fatigue; depression; anxiety **No**: physical exercise; cancer-related emotional stress; fear of progression	No between group analysis	Mean usage = 25.7 days (SD = 33.9) for three months intervention phase	Fair	
Hummel, 2017, The Netherlands	2.5 (mid treatment) 6.0 9.0 and 15.0 (intervention group only)	Sexual functioning, sexual distress	Body image, marital functioning, menopausal symptoms, psychological distress, quality of life	Hospital Anxiety and Depression Scale	**Yes**: sexual functioning (6 of 10 subscales); sexual distress; body image **No**: sexual functioning (4 of 10 subscales); marital functioning; menopausal symptoms; depression; anxiety; psychological distress; quality of life	No between group analysis	Not reported	Good	Only patients with sexual dysfunction
Oswald, 2021, USA	1.5 2.0	Breast cancer knowledge, coping, cancer-related self-efficacy	Not reported	Communication and Attitudinal Self-Efficacy scale for Cancer	**No**: breast cancer knowledge; coping; cancer-related self-efficacy	No long-term follow-up	Median usage = 63 min/week for 1.5 months intervention phase	Fair	Only Latina breast cancer survivors
van den Berg, 2015, The Netherlands	4.0 6.0 10.0	General psychological distress, general psychological empowerment	Negative adjustment (general and cancer-specific distress, fatigue, helplessness, and fear of cancer recurrence) and positive adjustment (self-efficacy, remoralisation, personal control, quality of life, fulfilment, re-evaluation, new ways of living, and valuing life)	Symptom Checklist 90, Hospital Anxiety and Depression Scale, Distress Thermometer, Cancer Worry Scale, Self-Efficacy Scale	**Yes:** distress; fatigue; fear of recurrence; cancer specific distress; general self-efficacy; general remoralisation; cancer-specific, new ways of living **No**: empowerment; distress thermometer; cancer specific helplessness; personal control; acceptance; perceived benefits; quality of life; fulfilment; re-evaluation; valuing life **2 months post intervention:** **Yes**: fear of cancer recurrence **No**: other primary and secondary outcomes	No significant intervention effect	Log-in frequency = 0-45; total usage duration = 0-2324 minutes	Good	
van Helmondt, 2019, The Netherlands	3.0 9.0	Fear of cancer recurrence	Coping strategies, functioning impairments, psychological distress, self‐efficacy, trait anxiety, (lack of) social support, depressive symptoms, physical problems	Psychosocial Distress Questionnaire-Breast Cancer, Dutch Fear of Cancer Recurrence Inventory	No significant intervention effect	No significant intervention effect	Not reported	Good	
Wagner, 2021, USA	1.0 2.0	Fear of cancer recurrence	Fear of cancer recurrence, cancer-specific distress, anxiety, depression, fatigue, sleep disturbance, cognitive problems, quality of life, self-efficacy	Impact of Events Scale, Patient-Reported Outcomes Measurement Information System, Breast Cancer Self-Efficacy Scale, Fear of Cancer Recurrence Inventory	No difference between the groups receiving intervention components and not receiving intervention components	No long-term follow-up	Average log-ins = 8.1 (SD = 8.27, range = 0–57)	Fair	Only patients reporting fear of cancer recurrence

Four trials reported time constraints of participants in the intervention group as reasons for dropout or low compliance [34, 37, 39, 40]. In three trials, participants dropped out because of the intervention not meeting the expectations and one trial reported technical problems as dropout-reason. One trial named patients’ health condition, problem with smartphone and distress as dropout reasons [39]. Three trials did not report dropout reasons.

Two studies did not report information about the usage of the mHealth intervention [41, 44]. One study reported adverse events related to intervention usage (increased distress (*N* = 8), reduction body weight (*N* = 1)) [42]. Two studies reported that usage time/frequency did not correlate with primary outcome [42, 43]. One study reported a more robust improvement in primary outcome for high users [36]. Two studies reported a higher use of mHealth intervention for the guided/telecoaching group compared to the self-guided group [36, 40]. One study reported that the degree of engagement with the app was not significantly associated with patients’ fear of cancer recurrence [39]. Five studies reported percentages of patients discontinuing the intervention: on average 8.3% of participants never started (range = 3–15%) and 23.4% discontinued the intervention earlier (range = 3–36%).

### Effects of psychological mHealth interventions on mental health of breast cancer survivors

#### Distress

Distress was measured in seven trials [34, 36–38, 40, 41, 43] (Table 2). Six different outcome measures were used: BSI-18, Dutch or NCCN distress thermometer, HADS total score, SCL-90 and Impact of Events Scale. Seven studies reported results up to 3 months after intervention, three of them with significant intervention effects [37, 41, 43]. Two studies reported long-term results (> 3 months post intervention) without significant intervention effects [40, 43].

#### Depression

Five trials reported depression as outcome using three different scales: HADS, PHQ-9, PROMIS [34, 36, 39, 40, 42]. All five trials assessed depression at short-term follow-up and one of the trials reported a significant intervention effect [42]. One trial assessed long-term effects (> 3 months post intervention), but no intervention effect was reported [40]. One trial additionally reported a significant within-group reduction of depressive symptoms from 2 to 6 months follow-up, but there was no 6 months follow-up assessment for the control group to compare the effect [39].

#### Anxiety and cancer-related fear

Five trials reported anxiety as outcome using three different scales: HADS, GAD-7, PROMIS [34, 36, 39, 40, 42]. All five trials assessed anxiety at short-term follow-up and one of the trials reported a significant intervention effect [42]. One trial assessed long-term effects (> 3 months post intervention), but no intervention effect was reported [40].

Additionally, cancer-related fear (fear of recurrence or progression) was reported by four trials as outcome using the scales PA-F12, CARS-J or FCRI [36, 39, 42, 44]. All of them assessed fear of recurrence at short-term follow-up (≤ 3 months post intervention). In one trial, an intervention effect was reported [39]. One trial measured fear of recurrence at the long-term and did not report an intervention effect [44].

#### Self-efficacy

Three trials reported self-efficacy as outcome using the scales CASC, SES and BSCE [35, 36, 43]. All three trials assessed self-efficacy at the short-term (≤ 3 months post intervention) and one of them reported a significant intervention effect [43]. Furthermore, this trial assessed self-efficacy at long-term follow-up, but there was no significant difference between the intervention and the control group [43].

## Discussion

### Main findings

In this systematic review, we identified eleven studies that examined the effectiveness of psychological mHealth interventions for breast cancer survivors. Regarding our predefined outcomes, we could not identify a clear significant effect of the psychological mHealth interventions. Possible reasons for the absence of an effect of the interventions are also discussed in previous studies. Low baseline levels of distress and, in some cases, high dropout rates could explain the results [38, 44]. Furthermore, no trial reported significant intervention effects on our predefined outcomes at long-term follow-up. However, long-term follow-up is important to assess the stability of intervention effects. Mental health impairments occur and may remain stable after completion of primary treatment, but intervention effects, e.g. for fear of cancer recurrence, are usually smaller with longer follow-up [3, 45, 46]. So, one could question whether patients can benefit from the intervention at all in the long term, but in many studies no long-term follow-up was carried out, which should be considered in future studies.

The wide heterogeneity of interventions and outcomes precluded the performance of a meta-analysis. The qualitative synthesis also reflects the existing heterogeneity: For the outcome distress, there was a tendency for a significant intervention effect (three of seven studies), for the outcomes depression, anxiety, fear of recurrence and self-efficacy, only one study each showed an effect, with two to four studies reporting no significant effect compared to control. A meta-analysis on telephone- and web-based interventions for breast cancer patients and survivors compared to routine treatment showed an intervention effect regarding better quality of life and sense of self-efficacy, lower scores for depression, distress and perceived stress, but not anxiety [15]. The lack of significant longer-term effects and the inconclusive results, which are also reflected in our study, could be due to the heterogeneity of populations and intervention targets. A systematic review of psychological eHealth interventions for cancer patients suggests that interventions well attuned to the needs of their participants were more efficient [16]. Thus, we also identified studies whose interventions targeted specific patient subgroups. They are therefore not easily summarised in terms of interventions, patients and outcomes.

### Study population

Six studies applied interventions specifically targeting patients with fatigue, hot flushes/night sweats, hormone receptor-positive cancer, sexual dysfunction, fear of cancer recurrence or Latina breast cancer survivors. Specific interventions assessed mental health outcomes mostly as secondary outcomes. The studies with the intervention for patients with hormone receptor-positive cancer and the intervention for fatigue reported a significant effect on mental health outcomes. The specific psychological interventions for hot flushes/night sweats, sexual dysfunction and fear of cancer recurrence showed no significant effect on mental health outcomes. However, significant results were reported for the primary outcomes related to the specific complaints for hot flushes/night sweats and sexual dysfunction. It can be assumed that the interventions are based on psychological models but target the specific symptoms and not general mental health. A high specificity of the interventions could explain why effects were mainly shown for the specific symptoms, but not for general mental health.

Therefore, the specific needs and symptoms of breast cancer patients can and should be given consideration in the interventions [3, 47]. However, there is also evidence that patients with different cancer diagnosis benefit from the same psychological eHealth intervention: A systematic review indicates that web-based services have potential for effective management of psychological distress, but study evidence is inconsistent [16]. The extent to which results from studies of general psychological mHealth interventions are applicable to (breast) cancer patients is unclear. In our review, we identified some studies that examined different cancer types or treatment stages and requested separate data for breast cancer survivors, but none were obtained. Trans-diagnostic psychological treatment approaches suggest that different patient groups may benefit from treatments [10, 12]. A systematic review of psychological eHealth interventions used before the onset of mental illness, directed at the healthy general population but also specific patient populations including cancer patients, reported promising effects of these interventions for the prevention of mental disorders [12]. For instance, general psychological distress is an additional burden to physical side effects of breast cancer survivors and can have far-reaching negative consequences for the disease management. These include an increase in the frequency and duration of hospitalisations, lower treatment adherence, difficulties in coping with the disease, reduced quality of life and an increase in mortality, leading to lower participation in working life and social life of those affected [48–54]. In our review, no association was found that patients with a higher symptom burden used the intervention more often, suggesting that support services are used regardless of the burden identified in the screening. Unfortunately, this was only investigated in one of the included trials, but it is consistent with preliminary results from another study in which patients sought psycho-oncological support independent of screening results, and with the generally high rate of breast cancer patients reporting unmet psychological needs [4, 5, 39, 55]. Due to the limited evidence available, future studies should consider possible patient subgroups and stress-related use of interventions and should facilitate subgroup analyses.

In general, the studies examined younger samples of breast cancer survivors [56]. Epidemiological data show that 80% of breast cancer patients are over 50 years of age, whereas in the included studies the mean age was on average 51.4 years (range = 43.9–56.2). This is likely due to the use of the web-based and mobile interventions and the frequent inclusion criteria of minimal technical skills or possession of a smartphone. However, it is also possible that (self-) selection bias occurred. Thus, the results might be more representative of a younger patient group with a minimum of technical knowledge.

### Intervention usage and effects

An important factor for mHealth interventions is the theoretical basis of application and content development. Only half of the included studies reported involving patients in the process of creating the mHealth intervention during development or in a pilot phase. However, a user-centred design is indispensable to ensure a fit between the service and users’ needs. Only in this way can the users benefit optimally from the offer. This is also the recommendation of user-centred and health-literature design for health communication and of the MRC guidelines for the development of complex interventions [13, 57]. The CONSORT-EHEALTH statement on reporting web-based and mHealth interventions recommends that the development process should be reported in the publication, which we would like to emphasise, because without this information, a comparison of interventions and replication is not possible [58]. Furthermore, the theory base in the development of application and content, as well as the inclusion of commercial providers, should be included in the qualitative assessment of studies in future reviews. The theoretical basis of all intervention content was CBT. It was not always clearly defined which CBT components were used, making a comparison of interventions difficult. A detailed description of the theory and applied components could be included in the supplement material, for example, and would be very important for comparability and implementation in practice. Trials with a significant effect based their applications generally on CBT or in detail on psychoeducation and stress management. Trials without a significant intervention effect on the defined mental health outcomes were also generally CBT-based or specifically applied coping and stress management.

Guidance, e.g. with regular contact with treatment providers via the applications, did not seem to influence the effectiveness of the interventions in our review. However, two studies that directly compared guided and non-guided interventions reported higher usage rates for the guided intervention, which is consistent with existing research on the relevance of guidance in motivating intervention use and potentially supporting intervention effectiveness [36, 40, 59, 60]. In general, however, higher usage time or frequency does not appear to be related to an effect on mental health outcomes, which was also shown in another study on mHealth interventions, where the intervention was effective for both the guided group with higher use and the self-help group with lower use [61]. While one study included in our review reported a more robust effect for high users, two other studies found no association [36, 42, 43]. The users of the intervention thus seem to be able to decide adequately on an individual basis whether they need the support provided by the application and whether they make use of it accordingly. However, it is unclear whether there is a possible lack of fit between the patients’ needs and the services offered in the intervention. Some study participants did not start the intervention or dropped out, for example, because of other expectations, lack of time or increased stress. It was rarely reported whether side effects were recorded or occurred. The most frequently mentioned was distress [42]. Future studies should focus on the reasons and barriers that discourage or motivate patients to use the applications. They should additionally identify which groups are not reached by the interventions to provide alternative services. Further, future studies should assess potential adverse effects, particularly in self-help services where adverse negative effects of interventions may not be well captured.

Additionally, intervention duration could be of importance. The interventions that were used in the included studies had a median duration of 3 months, ranging between 1 and 6 months. Interventions with a significant effect on our predefined outcomes had a duration of at least 2 months. Future studies should be as systematic as possible in the development and testing of their intervention to identify possible time effects.

### Strengths and limitations

The strength of our systematic review is the clear definition of the target population, namely breast cancer survivors who have completed primary therapy with a curative treatment approach in follow-up care. The definition of a psychological mHealth intervention was clearly specified in advance to reduce heterogeneity. Screening, extraction and assessing methodology and bias were always performed by two of a team of four reviewers.

However, several limitations should be noted. First, there are some psychological mHealth interventions that are aimed at no specific patient group or generally at cancer patients. These studies may not have been identified by our search. Further, emphasis was placed on psychological interventions that included at least one component such as psychoeducation or coping training to reduce heterogeneity between studies. However, applications that do not fall under this definition, such as exercise and activity promotion, may also have a positive impact on mental health [62]. These interventions were not included. Secondly, we only refer to breast cancer survivors. Also in the field of palliative care, studies are scarce and of course, this group, just like patients in primary care, has psychological support needs. Third, data extraction revealed that some patient-relevant outcomes were not included in the current analysis, such as fatigue, hot flushes/night sweats or sexual dysfunction. In particular, effects could be identified for some complaint-specific interventions in this regard.

Regarding the included studies, it should be noted that it was often not reported whether the protocol was followed exactly and whether other measures were used. However, this information is important to ensure a high-quality evaluation of the intervention for potential use in practice as well as possible replicability. Future studies should report these aspects if possible. Considering the technical development of the intervention, there are unfortunately still too few standards in the development of mHealth applications, which should always be taken into account when considering availability and comparability from a technical perspective. Future studies should follow harmonised standards to facilitate the screening of existing developments and evidence gaps and thus increase the quality of research [63]. Furthermore, previously reported reasons for dropout and patterns of use associated with the intervention should be taken into account when developing future interventions, and both should be reported in the studies. In addition, the effect sizes found so far should be used for power calculations to adequately plan and conduct further studies. The fact that some studies report similar improvements in outcomes in the intervention and control groups may also have methodological reasons that should be considered in future studies. A selection bias regarding a healthier population should be reduced by making access to digital services as low-threshold as possible. Since a heterogeneous population in terms of age and cancer diagnosis can benefit from mHealth self-help interventions, a broad group of patients should be targeted during recruitment [64]. Furthermore, care should be taken to select adequate scales for assessing the outcomes, which was the case in most of the included studies. Change-sensitive scales should be chosen that are also validated for use in a clinical sample [14, 65].

## Conclusion

The small number of trials finally included suggests that only few studies on psychological mHealth interventions specifically target breast cancer survivors. The studies available so far on psychological mHealth interventions specifically designed for or used by breast cancer survivors predominantly show no difference between the intervention and the control group. The few signs of possible intervention effects are generally not reproducible in all studies, so that future studies should investigate which patients in which setting benefit from the intervention. Therefore, psychological mHealth services for breast cancer survivors appear to be only a supportive component of treatment, and other psychological care should be provided accordingly to meet remaining needs.

## Supplementary Information

Below is the link to the electronic supplementary material.

Supplementary file1 (PDF 30 KB)

## Data Availability

Data sharing is not applicable to this article as no datasets were generated during the current study.
