# Supplementary material for: Systematic review on the effectiveness of mobile health applications on mental health of breast cancer survivors
Source: J Cancer Surviv. 2023 Oct 31;19(1):1–17. doi: 10.1007/s11764-023-01470-6 (PMC11814032; doi:10.1007/s11764-023-01470-6)
Supplement: Supplementary file 1 — Supplementary file1 (PDF 30 KB) [file 11764_2023_1470_MOESM1_ESM.pdf]

Supplement

Table S1. Search string for systematic search in databases.

MEDLINE via PubMed	("Breast Neoplasms"[MeSH Terms] OR "breast neoplasm*"[Title/Abstract] OR "breast cancer*"[Title/Abstract] OR "breast carcinoma"[Title/Abstract] OR "breast tumor*"[Title/Abstract]) AND ("Telemedicine"[MeSH Terms] OR "mHealth"[Title/Abstract] OR "eHealth"[Title/Abstract] OR "health app*"[Title/Abstract] OR "mobile application*"[Title/Abstract] OR "mobile phone*"[Title/Abstract] OR "smartphone*"[Title/Abstract] OR "medical app*"[Title/Abstract] OR "mobile health"[Title/Abstract] OR "digital intervention*"[Title/Abstract]) AND ("Follow-up care"[MeSH Terms] OR "Follow-up care"[Title/Abstract] OR "survivor*"[Title/Abstract]) AND ("Mental Health"[MeSH Terms] OR "Mental Health"[Title/Abstract] OR "mental disorder*"[Title/Abstract] OR "mental illness*"[Title/Abstract] OR "mood disorder*"[Title/Abstract] OR "depressi*"[Title/Abstract] OR "anxi*"[Title/Abstract] OR "self efficac*"[Title/Abstract] OR "self efficac*"[Title/Abstract] OR "psychological distress"[Title/Abstract] OR "psychological health"[Title/Abstract] OR "psychological disorder*"[Title/Abstract] OR "perceived stress"[Title/Abstract] OR "perceived distress"[Title/Abstract] OR "psychological stress"[Title/Abstract] OR "emotional adjustment*"[Title/Abstract] OR "adaptation, psychological"[MeSH Terms])
PsycInfo via ProQuest	(tiab("digital intervention*") OR tiab("mobile health") OR tiab("mobile appliction*") OR tiab("mobile application") OR tiab("mHealth") OR tiab("eHealth") OR tiab("online") OR tiab("health app") OR tiab("mobile phone") OR tiab("digital health app") OR tiab("mobile app") OR tiab("app") OR tiab("mobile phone app") OR tiab("mobile medical app") OR tiab("smartphone") OR tiab("medical app") OR tiab("information providing app") OR tiab("mobile-based") OR tiab("web-based") OR tiab("internet-based")) AND (tiab("Breast Neoplasm*") OR tiab("Breast Cancer*") OR tiab("breast tumor*") OR tiab("breast carcinom*")) AND (tiab("mental health") OR tiab("Self-effic*") OR tiab("Emotional Adjustment") OR tiab("Mental disorder*") OR tiab("mood disorder*") OR tiab("depressi*") OR tiab("Mental Illness") OR tiab("anxi*") OR tiab("self efficac*") OR tiab("psychological distress") OR tiab("psychological health") OR tiab("psychological disorder*") OR tiab("perceived stress") OR tiab("psychological stress") OR tiab("perceived distress") OR tiab("emotional adjustment")) AND (tiab("survivor*") OR tiab("follow-up care"))
Cochrane Database of Systematic Reviews and Cochrane Central Register of Controlled Trials via Cochrane Library	#1 ("breast cancer"):ti,ab,kw<br>#2 MeSH descriptor: [Breast Neoplasms] explode all trees<br>#3 ("breast tumor*"):ti,ab,kw<br>#4 ("breast tumour*"):ti,ab,kw<br>#5 ("breast neoplasm*"):ti,ab,kw<br>#6 ("breast carcinoma*"):ti,ab,kw<br>#7 #1 OR #2 OR #3 OR #4 OR #5 OR #6<br>#8 ("health app*"):ti,ab,kw<br>#9 (mhealth):ti,ab,kw<br>#10 (ehealth):ti,ab,kw<br>#11 ("mobile app*"):ti,ab,kw<br>#12 ("mobile phone"):ti,ab,kw<br>#13 (smartphone*):ti,ab,kw<br>#14 ("medical app*"):ti,ab,kw<br>#15 ("mobile health"):ti,ab,kw<br>#16 ("digital intervention*"):ti,ab,kw<br>#17 MeSH descriptor: [Telemedicine] explode all trees<br>#18 #8 OR #9 OR #10 OR #11 OR #12 OR #13 OR #14 OR #15 OR #16 OR #17

	#19	(follow-up care):ti,ab,kw
	#20	(survivor*):ti,ab,kw
	#21	MeSH descriptor: [Follow-up care] explode all trees
	#22	#19 OR #20 OR #21
	#23	("mental health"):ti,ab,kw
	#24	(depressi*):ti,ab,kw
	#25	(anxi*):ti,ab,kw
	#26	MeSH descriptor: [Mental Health] explode all trees
	#27	("mental disorder*"):ti,ab,kw
	#28	("mental illness*"):ti,ab,kw
	#29	("mood disorder*"):ti,ab,kw
	#30	("self efficacy"):ti,ab,kw
	#31	("psychological distress"):ti,ab,kw
	#32	("psychological stress"):ti,ab,kw
	#33	("perceived stress"):ti,ab,kw
	#34	("perceived distress"):ti,ab,kw
	#35	("psychological health"):ti,ab,kw
	#36	("psychological disorder*"):ti,ab,kw
	#37	("emotional adjustment'*"):ti,ab,kw
	#38	MeSH descriptor: [Adaptation, Psychological] explode all trees
	#39	#23 OR #24 OR #25 OR #26 OR #27 OR #28 OR #29 OR #30 OR #31 OR #32 OR #33 [illegible] #34 OR #35 OR #36 OR #37 OR #38
	#40	#7 AND #18 AND #22 AND #39
PROSPERO	#1	"breast neoplasm*"
	#2	"breast cancer*"
	#3	"breast carcinoma*"
	#4	"breast tumor*"
	#5	MeSH DESCRIPTOR Breast Neoplasms EXPLODE ALL TREES
	#6	MeSH DESCRIPTOR Telemedicine EXPLODE ALL TREES
	#7	mhealth
	#8	ehealth
	#9	"mobile app*"
	#10	"health app*"
	#11	"medical app*"
	#12	"smartphone*"
	#13	"mobile phone*"
	#14	"mobile health"
	#15	"digital intervention*"
	#16	"breast tumour*"
	#17	follow-up care
	#18	survivor*
	#19	MeSH DESCRIPTOR Follow-up care EXPLODE ALL TREES
	#20	MeSH DESCRIPTOR Mental Health EXPLODE ALL TREES
	#21	"mental health"
	#22	"mental disorder*"
	#23	"mental illness*"
	#24	"mood disorder*"

	#25	"depressi*"
	#26	"anxi*"
	#27	"self-efficac*"
	#28	"self efficac*"
	#29	"psychological stress"
	#30	"psychological distress"
	#31	"perceived distress"
	#32	"perceived stress"
	#33	"psychological health"
	#34	"psychological disorder*"
	#35	"emotional adjustment*"
	#36	"psychological adaptation*"
	#37	#1 OR #2 OR #3 OR #4 OR #5 OR #16
	#38	#6 OR #7 OR #8 OR #9 OR #10 OR #11 OR #12 OR #13 OR #14 OR #15
	#39	#17 OR #18 OR #19
	#40	#20 OR #21 OR #22 OR #23 OR #24 OR #25 OR #26 OR #27 OR #28 OR #29 OR #30 O[illegible] #31 OR #32 OR #33 OR #34 OR #35 OR #36
	#41	#37 AND #38 AND #39 AND #40
